# Specialism

**Published:** 1899-07-01

**Authors:** 


### SPECIALISM.

The advantages and the disadvantages of specialism were pretty fully discussed at the recent meeting of the American Academy of Medicine. We cannot see that any very fresh ground was broken, but what was made abundantly evident was that it is becoming generally recognised that the range of topics embraced in what is termed "medicine" is far too great to be thoroughly mastered by any one mind, and that it is only by specialism that satisfactory progress can be made. It was, however, not uninteresting to observe that speaker after speaker pointed out to how large an extent the evils of specialism are aggravated by, and, in fact, are primarily due to, the growing custom of young men taking up specialities "from the beginning," instead of first becoming skilful all-round physicians and specialising afterwards, as their opportunities or their inclinations lead to this or that field of research. This and the love of money are indeed the real causes of most of the evils which make the word "specialism" stink in the nostrils of the general practitioner. If the specialist could but take as wide a view as the general practitioner and contribute some special knowledge in addition, then, indeed, he would be worth his salt; for notwithstanding the overweening sense of his own importance with which he is too often oppressed, and the tendency he too often shows to hide his thoughts in curiously obscure language comprehended only by the priesthood in his own particular cult, the specialist is after all a necessity of the times, and ought to represent, as we think he often does, the growing-point of knowledge in his own special line of work.

